# Bone Health in Rats With Temporal Lobe Epilepsy in the Absence of Anti-Epileptic Drugs

**DOI:** 10.3389/fphar.2019.01278

**Published:** 2019-10-29

**Authors:** Rhys D. Brady, Ker Rui Wong, Dale L. Robinson, Richelle Mychasiuk, Stuart J. McDonald, Ryan A. D’Cunha, Glenn R. Yamakawa, Mujun Sun, John D. Wark, Peter Vee Sin Lee, Terence J. O’Brien, Pablo M. Casillas-Espinosa, Sandy R. Shultz

**Affiliations:** ^1^Department of Neuroscience, Central Clinical School, Monash University, Melbourne, VIC, Australia; ^2^Department of Medicine, The Royal Melbourne Hospital, The University of Melbourne, Parkville, VIC, Australia; ^3^Department of Biomedical Engineering, University of Melbourne, Parkville, VIC, Australia; ^4^Department of Physiology, Anatomy, and Microbiology, La Trobe University, Bundoora, VIC, Australia; ^5^School of Medicine, Deakin University, Geelong, VIC, Australia

**Keywords:** bone, epilepsy, status epilepticus, animal model, micro-computed tomography

## Abstract

**Rationale:** Epilepsy patients often exhibit reduced bone mineral density and are at an increased risk of bone fracture. Whether these bone abnormalities are due to the use of anti-epileptic drugs (AED’s) or the disease itself is unknown. For example, although decreased bone health in epilepsy patients is generally attributed to the use of AED’s, seizures can also trigger a number of physiological processes that have the potential to affect bone. Therefore, to assess whether bone abnormalities occur in epilepsy in the absence of AED’s, the current study investigated mechanical characteristics and trabecular bone morphology in rats with chronic temporal lobe epilepsy.

**Methods:** Ten-week old male Wistar rats underwent kainic acid-induced status epilepticus (SE; n = 7) or a sham procedure (n = 9). Rats were implanted with EEG recording electrodes at nine weeks post-SE, and video-EEG was continuously recorded for one week at 10- and 22-weeks post-SE to confirm that SE rats had spontaneous seizures. Open-field testing to assess locomotion was conducted at 23-weeks post-SE. At 24-weeks post-SE, rats were euthanized and tibia were extracted to determine trabecular morphology by micro-computed tomography (µCT), while femurs were used to investigate mechanical properties *via* 3-point bending.

**Results:** All post-SE rats had spontaneous seizures at 10- and 22-weeks post-SE, while none of the sham rats had seizures. µCT trabecular analysis of tibia revealed no differences in total volume, bone volume, bone volume fraction, trabecular number, or trabecular separation between post-SE or sham rats, although post-SE rats did have increased trabecular thickness. There were also no group differences in total distance travelled in the open field suggesting that activity levels did not account for the increased trabecular thickness. In addition, no differences in mechanical properties of femurs were observed between the two groups.

**Conclusion:** There was a lack of overt bone abnormalities in rats with chronic temporal lobe epilepsy in the absence of AED treatment. Although further studies are still needed, these findings may have important implications towards understanding the source (e.g., AED treatments) of bone abnormalities in epilepsy patients.

## Introduction

Epilepsy is a complex group of neurological disorders that affects ∼50 million people worldwide. The disease is defined as when the brain demonstrates an enduring tendency to have recurrent seizures (Fisher et al., 2014). Epilepsy is associated with several co-morbidities; however, a commonly underappreciated consequence of the condition is impairment in bone health (i.e., reduced bone quantity and/or impaired bone quality) (Petty et al., 2016). Indeed, numerous studies have reported that patients with epilepsy have an increased risk of bone fractures both during seizures and also in the absence of seizures, (Vestergaard et al., 2004; Nakken and Taubøll, 2010) which is in part due to the reduced bone health commonly observed in these patients (Petty et al., 2016).

Reduced bone health and increased risk of fracture are often attributed to the use of antiepileptic drugs (AED’s) (Vestergaard, 2015; Rahimdel et al., 2016). The majority of epilepsy patients are treated with AED’s and there is some evidence that certain AED’s may be associated with reduced bone health (e.g., levetiracetam, (Nissen-Meyer et al., 2007; Aksoy et al., 2016;Hakami et al., 2016) oxcarbazepine, (Aksoy et al., 2016) sodium valproate, (Sato et al., 2001; Nissen-Meyer et al., 2007) and phenytoin, (Välimäki et al., 1994;Nissen-Meyer et al., 2007), while others have yet to be rigorously studied. However, whether these bone abnormalities are due to the use of anti-epileptic drugs (AED’s) or the disease itself remains unknown (Petty et al., 2016). It is important to consider that seizures can trigger a number of central and systemic physiological processes that have potential to affect bone health. Therefore, it is possible that recurrent spontaneous seizures contribute to the reduced bone health observed in patients with epilepsy (Petty et al., 2016). For example, during and after seizures there is a period of sympathetic hyperactivity (Devinsky, 2004; Poh et al., 2012; Picard et al., 2017).This increased sympathetic outflow has potential to increase activation of β2 adrenergic receptors on osteoblasts (i.e., bone forming cells) (Kondo et al., 2012). The activation of these osteoblastic receptors has been found to stimulate osteoclast induced bone resorption (Kondo et al., 2012). Further, seizures induce an inflammatory cascade that is characterized by up-regulation of pro-inflammatory cytokines and chemokines, and the activation and migration of immune cells (Kumar and Loane, 2012). The disruption of the blood brain barrier (BBB) that occurs during and following seizures (Librizzi et al., 2012; Van Vliet et al., 2015) may facilitate the migration of these inflammatory mediators from the brain into the peripheral circulation where they could influence bone metabolism (Van Vliet et al., 2015). In particular, it has been well established that increased systemic inflammation is capable of activating osteoclasts, triggering bone resorption (Lee et al., 2008; Ciucci et al., 2015). Furthermore, the generation and migration of oxidative stress mediators during/post seizures may suppress bone formation and promote osteoclastic differentiation (Garrett et al., 1990; Kondo et al., 2012; Williams et al., 2015).

It is difficult to study the effect of recurrent spontaneous seizures on bone health in humans as most patients are treated with AED’s and also present with a number of confounding factors (e.g., comorbidities, socioeconomic/lifestyle factors) (Petty et al., 2016). In addition, changes in bone mass in patients with epilepsy take years to manifest (El-Hajj Fuleihan et al., 2008; Nakken and Taubøll, 2010). Animal models of epilepsy allow for rigorous investigation of the effect of seizures on bone in a time- and cost-efficient manner (Brady et al., 2018). Therefore, the aim of this study was to examine the effect of recurrent spontaneous seizures on the quality and quantity of the tibia in a rat model of chronic temporal lobe epilepsy.

## Methods

### Subjects

Ten-week old male Wistar rats were bred and housed in the Department of Medicine, University of Melbourne Biomedical Research Facility. Rats were housed individually under a 12 h light/dark cycle and given access to food and water *ad libitum* for the duration of the experiment. All experimental procedures were approved by The Florey Animal Ethics committee (Ethics number: 16-047 UM).

### Kainic Acid-Induced Post-Status Epilepticus

Rats were randomly assigned to receive either sham (n = 9) or kainic acid (KA)-induced status epilepticus (SE; n = 7). The post-SE rat model of temporal lobe epilepsy is well characterized and mimics the epileptogenic processes observed in humans (Van Nieuwenhuyse et al., 2015). A repeated low dose KA administration protocol modified from Hellier and colleagues was used (Hellier et al., 1998; Bhandare et al., 2017). Rats in the SE group were given an i.p. injection of 7.5 mg/kg KA in 3 ml of saline, while shams were injected with saline only. Rats were subsequently monitored for behavioural seizures as assessed *via* the Racine scale. Briefly, the Racine scale categorises seizure severity into 5 classes: Class I is defined by mouth and facial movements; Class II by head nodding; Class III by forelimb clonus; Class IV by bilateral forelimb clonus and rearing; Class V by rearing and falling (Racine, 1972). If no self-sustained seizure activity was observed (i.e., at least five class IV Racine scale seizures), another i.p. dose of 2.5 mg/kg of KA was administered up to a maximum of 15 mg/kg. Rats were excluded from the experiment if they did not show a stable self-sustained SE after the maximum KA dose. After 4 h of sustained behavioral seizures the rats were given diazepam (5 mg/kg/dose) to stop the SE.

### Electrode Implantation

Rats were implanted with EEG recording electrodes at 9-weeks post-SE under isoflurane induced anaesthesia. Each rat received a subcutaneous injection of carprofen analgesic (5 mg/kg; Rimadyl; Pfizer Australia). Six burr holes were drilled through the skull. relative to bregma, one electrode was positioned at each of the following six co-ordinates: I) AP+2.0mm; II) AP-2.0mm; III) AP-4.5mm, ML+2.5mm; IV) AP-4.5mm, ML-2.5mm; V) AP-8.0mm, ML+2.0mm; and VI) AP-8.0mm, ML-2.0mm. the recording electrodes were embedded by applying dental cement around the electrodes and over the skull.

### Video-EEG Recordings and Seizure Analysis

As previously described, (Shultz et al., 2013; Liu et al., 2016; Casillas-Espinosa et al., 2019a; Santana-Gomez et al., 2019) rats underwent video-EEG recordings continuously (i.e., 24 h/day) for one week at 10- and 22-weeks post-SE. Video-EEG recordings were obtained using Profusion 3 software (Compumedics, Australia) unfiltered and digitized at 512 Hz. EEG analysis was performed by an investigator blinded to the experimental groups. All EEG recordings were screened for seizures using automated software (Assyst, Australia) (Casillas-Espinosa et al., 2019a). Seizure events were visually confirmed using Profusion 3 software. A seizure was defined as an episode of rhythmic spiking activity that was three times the baseline amplitude and a frequency > 5 Hz that lasted at least 10 s (Pitkänen et al., 2005; Van Nieuwenhuyse et al., 2015; Liu et al., 2016; Casillas-Espinosa et al., 2019b). The end of a seizure was determined as the last spike. The average number of seizures per day, average seizure duration and seizure class (i.e. severity) were analysed.

### Open-Field Testing

At 23 weeks post-SE, locomotion was assessed using an open-field as previously described (Shultz et al., 2013; Shultz et al., 2014). Rats were placed in the centre of a circular open-field arena (100 cm diameter) enclosed by walls 20 cm high, and allowed to freely explore for 5 min. Behaviour in the open-field was recorded by an overhead camera, and *Ethovision Tracking Software* (Noldus, Netherlands) quantified the total distance travelled as well as the number of entries and time spent in the centre area (66 cm diameter) of the arena.

### μCT

Rats were euthanized at 24-weeks post-SE and the right tibia was extracted and fixed in 4% paraformaldehyde for 24 h, then washed in PBS and stored in 70% ethanol at 4°C (Brady et al., 2014; Brady et al., 2016a; Brady et al., 2016b; Brady et al., 2016c;). Before scanning, tibia were rehydrated in 0.9% saline solution for 14 h to avoid any changes in medullary density in the trabecular region. Images were acquired using a Scanco μCT 50 scanner (Scanco Medical AG, Switzerland), with a tube voltage, current and integration time of 70 kV, 144 μA and 300 ms, respectively, and isotropic voxels of 10 μm (Stauber and Müller, 2008; Schambach et al., 2010). A 0.5 mm aluminium filter was used to reduce beam hardening artefacts, two scanning iterations were used to reduce noise and tibia specimens were immersed in saline solution during scanning to prevent dehydration (Irie et al., 2018).

The region of interest (ROI) was designated as being a 2.5 mm region, beginning 1 mm from the growth plate and extending distally (Nishiyama et al., 2010; Campbell et al., 2011; Campbell and Sophocleous, 2014; Liu et al., 2015). Trabecular bone morphology within the ROI was computed using the evaluation scripts available in the Scanco IPL software (v6.1, Scanco Medical AG, Switzerland) with the following settings: threshold 220–1,000, Gaussian noise filter: Sigma 0.8, support 1. Trabecular bone parameters computed included: Total volume (TV), bone volume (BV), bone volume fraction (BV/TV), connectivity density (Conn.D), trabecular number (Tb.N), trabecular thickness (Tb.Th), and trabecular separation (Tb.Sp).

### Mechanical Testing

Biomechanical properties of the diaphysis of the right femur (mediolateral bending) were compared between sham and post-SE rats at 24-weeks post-SE using a three-point bending apparatus (Brady et al., 2016a; Brady et al., 2016c; Leppanen et al., 2006). Load and deflection data were recorded continuously using transducers connected to an x–y plotter by preamplifiers. Peak force to failure and stiffness were calculated from the load deflection data.

### Statistical Analyses

Statistical analyses of data were performed using IBM SPSS Statistics version 25 (Armonk, New York, USA). An independent-samples t-test was used to compare bone parameters in both experimental groups. Statistical significance was set at p < 0.05.

## Results

### Video-EEG Recordings Analysis

At 10-weeks post-SE, post-SE rats averaged approximately 1 seizure per day with a mean seizure duration of 57 s and a mean seizure severity of 4.8, assessed *via* the Racine scale (see **Table 1**). At 22-weeks post-SE, rats 3 seizures per day, average duration of 58 s and a mean seizure severity of 4.8. No seizures were observed in shams rats at either recovery time.

**Table 1:** Seizure analysis in shams and post-SE rats.

		Week 10 post-SE			Week 22 post-SE		
		Seizures/day	Seizure severity	Seizure duration (s)	Seizures/day	Seizure severity	Seizure duration (s)
SHAM *n = 9*	Mean ± SEM	0 ± 0	0 ± 0	0 ± 0	0 ± 0	0 ± 0	0 ± 0
Post-SE *n = 7*	Mean ± SEM	1.09 ± 0.50	4.81 ± 0.12	57.4 ± 6.47	3.02 ± 1.07	4.83 ± 0.09	58.3 ± 3.73

All post-SE rats had spontaneous seizures at 10- and 22-weeks. There was no evidence that sham rats had spontaneous seizures at either of the recovery times.

### Locomotor Activity

**Figure 1:**
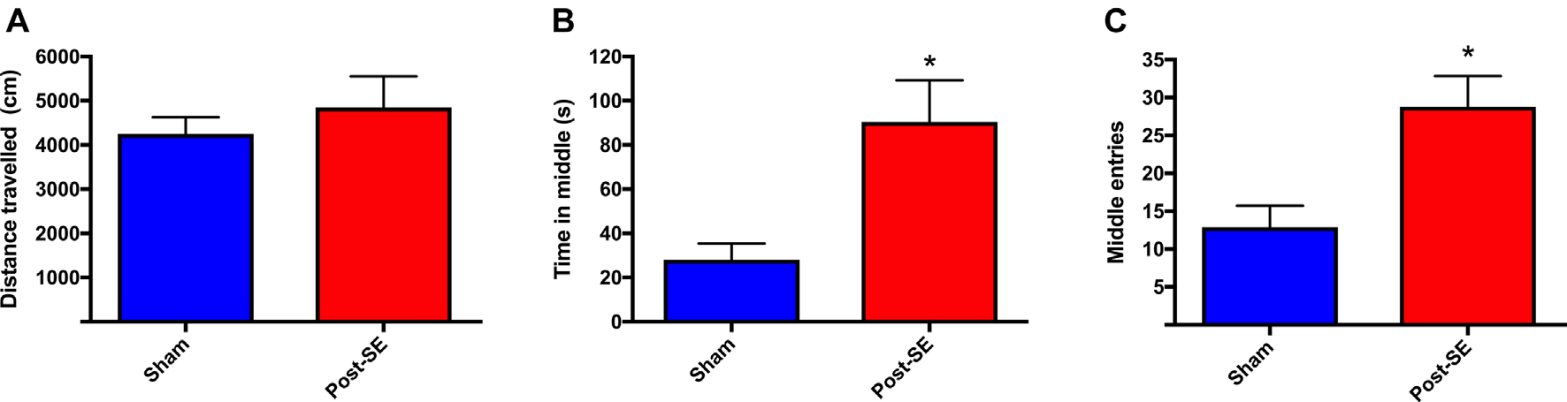
Distance travelled in an open-field. No difference in total distance travelled during open-field session was observed between groups **(A)**. Post-SE rats spent more time in the middle **(B)** and had significantly more middle entries **(C)** when compared to sham. Bars represent means ± SEM and * represents p < 0.05.

Locomotor activity was assessed at 23 weeks post-SE in the open-field. There were no differences in distance travelled in the open-field between sham and post-SE rats (**Figure 1**). However, post-SE rats spent more time in the middle of the arena, and had significantly more middle entries when compared to sham rats (p < 0.05).

### µCT Analysis

**Figure 2:**
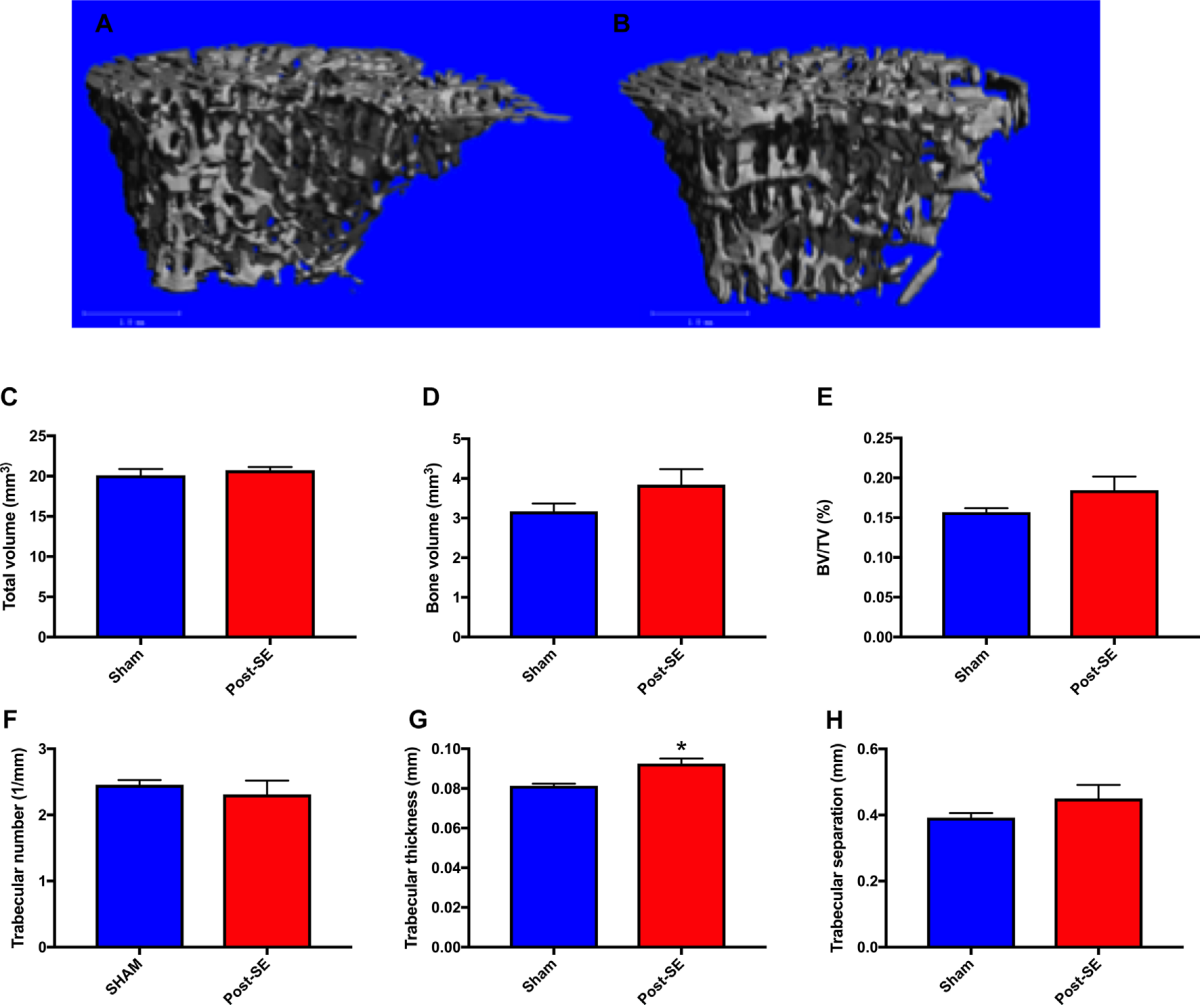
Representative μCT reconstructions of trabecular bone regions from sham **(A)** and Post-SE rats **(B)**. There were no differences between post-SE rats when compared to shams on the measures of total volume **(C)**, bone volume **(D)**, bone volume fraction **(E)**, trabecular number **(F)** or trabecular separation **(H)**. However, trabecular thickness **(G)** was significantly increased in post-SE rats when compared to shams. Bars represent means ± SEM and * represents p < 0.05.

µCT analysis revealed no between-group differences in total volume, bone volume, bone volume fraction, trabecular number, or trabecular separation between post-SE or sham rats. There was however, a significant increase in trabecular thickness in SE rats compared to shams (**Figure 2** g; p < 0.05).

### Mechanical Testing

No differences in peak force to failure or stiffness was observed between femora from post-SE rats when compared to shams (see **Table 2**).

**Table 2:** Mechanical characteristics of femora from sham and post-SE rats.

Group	Peak Force (N)	Stiffness (x 10^4^ Nm^2^)
Sham n = 9	407.2 ± 14.73	76.21 ± 4.50
Post-SE n = 7	422.3 ± 26.97	77.97 ± 7.48

There were no differences in mechanical properties at between sham and post-SE groups. Values are means ± SEM.

## Discussion

Few studies have investigated bone health in epilepsy in the absence of AED’s. Therefore, here the microarchitecture of the tibia and biomechanical properties of the femur were assessed in a rat model of chronic temporal lobe epilepsy without AED treatment. SE rats averaged one seizure/day at 10 weeks post-SE and three seizures/day at 22 weeks post-SE, whereas there was no evidence of seizures in sham rats. µCT analysis revealed that there were no overt bone microstructural abnormalities present in SE rats when compared to shams, as evidenced by a lack of differences in trabecular bone parameters including: total volume, bone volume, bone volume fraction, trabecular number or trabecular separation. Although there was a subtle increase in trabecular thickness in post-SE rats, this is not considered a marker of decreased bone health (Nilsson et al., 1986; Fonseca et al., 2014). No differences were observed in mechanical properties of the femoral midshaft. It is possible that ambulatory state could confound bone outcomes, as increased mechanical loading of bone is associated with bone formation, while reduced loading can trigger bone resorption (Vicente-Rodríguez et al., 2005). However, in this study, and others, (Inostroza et al., 2012) SE rats did not display a prolonged decrease in locomotion, suggesting that decreased activity levels did not account for the subtle increase in trabecular thickness. Taken together, the present findings suggest that this experimental model of acquired epilepsy does not cause changes in bone morphological parameters that may be detrimental to bone health.

Changes in bone mass in patients with epilepsy typically occur over a 1–5 year period, (El-Hajj Fuleihan et al., 2008; Nakken and Taubøll, 2010) although, this can differ due to a number of factors (e.g., medication, age, gender, type of epilepsy, and nutritional status). (Ahmad et al., 2017) Therefore, it is possible that decreased bone health in the SE rats may take longer to manifest and may have occurred at time-points not featured in this study. However, when one takes into account the life-span of the rat (1–4 years) and that the SE rats had evidence of severe seizures for > 5 months, it appears unlikely that significant changes would have occurred at more chronic stages. Moreover, our finding that trabecular thickness was actually increased in post-SE rats may further indicate that post-SE rats were unlikely to have bone loss at a later stage. Further studies examining gene and protein expression, are required to determine the precise mechanism through which post-SE rats had increased trabecular thickness and its potential biological significance. However, several studies have reported that serum levels of leptin are significantly increased following amygdala electrical kindling (a model of temporal lobe epilepsy) in rats (Bhatt et al., 2004; Bhatt et al., 2005; Hum et al., 2009). Given that peripherally-acting leptin stimulates bone formation it is possible that leptin may have played a role in the increased trabecular thickness observed in the current study (Reseland et al., 2001; Gordeladze et al., 2002; Wei et al., 2008; Wang et al., 2011). The administration of kainic acid may have influenced bone metabolism. For example, treatment of osteoclasts with 10–100 µM of NBQX a kainic acid receptor antagonist decreased osteoclastic bone resorption *in vitro* (Szczesniak et al., 2005). However, the exact effect that kainic acid treatment had on bone in this study requires further investigation. It is also possible that the increased trabecular thickness may have been due to increased loading caused by locomotion in post-SE rats. However, the current study and others (Inostroza et al., 2012), found no differences in distance travelled between post-SE rats and shams. These findings suggest that that the lack of overt bone abnormalities between the two groups was not due to differences in mechanical loading of the bones, which may influence bone volume and thickness. However, it is important to consider that although we observed no differences in locomotion between the two groups, it is possible that post-SE rats were more active at time-points not featured in this study. Furthermore, it should be noted that some studies have reported that post-SE rats travel further in the open field, (Petkova et al., 2014) while other studies have reported post-SE rats travel less in the open field (Yu et al., 2018). Future studies would benefit from monitoring physical activity of all rats in their home-cages to directly determine activity levels of sham and post-SE rats.

This study also found no differences in biomechanical properties of the femoral midshaft between sham and post-SE rats. It is likely that this is due to the slow remodelling rate of this region that is comprised predominantly by cortical bone (Fonseca et al., 2014). When compared with trabecular bone, cortical bone has a much lower surface-to-volume ratio and hence remodels at a much lower rate (Szulc and Seeman, 2009). Accordingly, changes in bone parameters typically manifest first in trabecular bone. Taken together with the absence of changes in trabecular bone microstructure, it is unlikely that changes occurred in bones not featured in this study. However, future studies should examine both trabecular and cortical bone microstructure, mechanical characteristics at various locations (e.g., tibia, femur and vertebrae), as well as blood-based markers of bone turnover to enhance the understanding of the effect of seizures on bone. Examination of bone mineral content and collagen cross-linking *via* Fourier transform infrared microspectroscopy, as was done in the initial Garip studies, (Szulc and Seeman, 2009; Rolvien et al., 2017) would also be informative.

A recent study used Wistar Albino Glaxo/Rijswijk (WAG/Rij) rats, a polygenic rat model of genetic generalised epilepsy with absence seizures, and assessed changes in bone mineral content and mineral matrix ratios following a 5-week audiogenic kindling regime (Garip et al., 2013). Fourier transform infrared microspectroscopy (FTIRM) analysis revealed that rats with epilepsy had reduced mineral content and collagen crosslinks, while B type carbonate was increased (Garip et al., 2013). The aforementioned findings have all been associated with reduced biomechanical properties and suggest that seizures in WAG/Rij rats may compromise bone health (Garip et al., 2013). However, WAG/Rij rats which, have an unknown inherited mutation, (Garip et al., 2013) were compared to healthy Wistar rats. Given that genetic mutations that result in seizures have previously been associated with bone loss and skeletal fragility, (Rolvien et al., 2017) it is unclear whether the bone abnormalities observed in WAG/Rij rats were due to the seizures or the unknown inherited mutation (Rolvien et al., 2017; Garip Ustaoglu et al., 2018).

In a follow-up study by the same group, however, WAG/Rij rats that had seizures induced by audiogenic kindling were compared to WAG/Rij rats that did not experience seizures when exposed to the same stimuli (Garip Ustaoglu et al., 2018). Following 5-weeks of kindling, rats that had seizures displayed reduced mineral and matrix properties in cortical and trabecular bone regions of the tibia, femur and spine when compared to rats that did not have seizures, suggesting that the seizures induced abnormalities in bone (Garip Ustaoglu et al., 2018). Furthermore, it was also demonstrated that treatment with the AED carbamazepine reduced mineral and matrix properties in cortical and trabecular regions of the tibia, femur and spine compared to rats that were not susceptible to audiogenic kindling (Garip Ustaoglu et al., 2018). The difference in findings between the current study and work by Garip et al. is uncertain but, may be due to differences in seizure mechanisms which cause non-convulsive absence seizures induced by audiogenic kindling and the tonic clonic recurrent spontaneous seizures that occur in the post-SE model of acquired epilepsy. Additionally, a potential limitation of FTIRM is that in the context of analysing bone it cannot be performed *in vivo* (Rolvien et al., 2017; Garip Ustaoglu et al., 2018). Therefore, although it is unlikely that there is a link between audiogenic seizure susceptibility and bone mineral content, to further demonstrate that the changes observed were due to seizures, a longitudinal study is required to confirm that WAG/Rij rats that have audiogenic seizures do not have reduced mineral content at baseline compared to those that do not. Although this study and others have provided insight into the effect of epilepsy on bone health, a limitation of this work so far is that only male rats have been studied. Evidence suggests that, post-menopause, females with epilepsy may have an increased risk of developing bone abnormalities (Ensrud et al., 2004; Lyngstad-Brechan et al., 2008). Therefore, it would be beneficial to examine bone health in aged or ovariectomized female rats (post-menopausal rodent models) with epilepsy. An additional limitation of this study is that µCT scans were performed post-mortem, hence there were no pre-seizure baseline measurements of bone. Further studies utilising serial *in vivo* µCT analysis pre-SE and at multiple time-points post-SE to assess changes in bone growth and morphology overtime may provide further insight how epilepsy may affect bone.

Several human studies have reported that AED’s are associated with reduced bone health, particularly in patients with acquired temporal epilepsy. For example, levetiracetam, (Nissen-Meyer et al., 2007; Aksoy et al., 2016; Hakami et al., 2016) oxcarbazepine, (Aksoy et al., 2016) sodium valproate, (Sato et al., 2001; Nissen-Meyer et al., 2007) and phenytoin, (Välimäki et al., 1994; Nissen-Meyer et al., 2007) have been associated with bone loss in patients with epilepsy (Beniczky et al., 2012; Aksoy et al., 2016; Hakami et al., 2016). In addition to AED’s, patients with epilepsy often have a lack of exposure to sunlight, limited physical activity and a high prevalence of vitamin D deficiency which all contribute to reduced bone health (Beerhorst et al., 2013). Furthermore, patients with epilepsy have an increased risk of seizure related trauma (e.g., slips and falls) which increases the likelihood of fracture (Beerhorst et al., 2013; Petty et al., 2016). The novel findings of the current study suggest that rats with chronic acquired temporal lobe epilepsy in the absence of AED’s do not have overt changes in bone morphological parameters, or mechanical properties indicative of decreased bone health. Whilst the results suggest that the bone loss observed in patients with acquired temporal epilepsy may be due to the use of AED’s, other comorbidities, or socioeconomic/lifestyle factors, future studies are still required to determine the cause of bone deficiencies in epilepsy patients and how they can be prevented.

## Data Availability Statement

All datasets generated for this study are included in the article/supplementary material.

## Ethics Statement

The animal study was reviewed and approved by The Florey Animal Ethics committee (Ethics number: 16-047 UM).

## Author Contributions

All authors contributed to the writing of the manuscript. RB, PC-E, TO’B, RM, and SS conceptualized and designed the experiments. PC-E completed the seizure analysis. KW, DR, RB, and PL completed the µCT scanning and analysis.

## Funding

This work was supported by grants to SS, and T O’B, from the NHMRC, and a Monash University interdisciplinary research grant to RB.

## Conflict of Interest

The authors declare that the research was conducted in the absence of any commercial or financial relationships that could be construed as a potential conflict of interest.
